# Muscle MRI in Patients With Oculopharyngeal Muscular Dystrophy: A Longitudinal Study

**DOI:** 10.1212/WNL.0000000000207833

**Published:** 2023-12-14

**Authors:** Rosemarie H.M.J.M. Kroon, Johanna G. Kalf, Bert J.M. de Swart, Linda Heskamp, Jacky W.J. de Rooy, Baziel G.M. van Engelen, Corinne G.C. Horlings

**Affiliations:** From the Departments of Rehabilitation (R.H.M.J.M.K., J.G.K., B.J.M.d.S.) and Neurology (B.G.M.v.E., C.G.C.H.), Radboud University Medical Center, Donders Institute for Brain, Cognition and Behaviour, Nijmegen; Department of Radiology (L.H.), University Medical Centre Utrecht; Department of Imaging (J.W.J.d.R.), Radboud University Medical Center, Nijmegen; and Department of Neurology (C.G.C.H.), Medical University of Innsbruck, Austria.

## Abstract

**Background and Objectives:**

Oculopharyngeal muscular dystrophy (OPMD) is a rare progressive neuromuscular disease. MRI is one of the techniques that is used in neuromuscular disorders to evaluate muscle alterations. The aim of this study was to describe the pattern of fatty infiltration of orofacial and leg muscles using quantitative muscle MRI in a large national cohort and to determine whether MRI can be used as an imaging biomarker of disease progression in OPMD.

**Methods:**

Patients with OPMD (18 years or older) were invited from the national neuromuscular database or by their treating physicians and were examined twice with an interval of 20 months, with quantitative MRI of orofacial and leg muscles to assess fatty infiltration which were compared with clinical measures.

**Results:**

In 43 patients with genetically confirmed OPMD, the muscles that were affected most severely were the tongue (mean fat fraction: 37.0%, SD 16.6), adductor magnus (31.9%; 27.1), and soleus (27.9%; 21.5) muscles. The rectus femoris and tibialis anterior muscles were least severely affected (mean fat fractions: 6.8%; SD 4.7, 7.5%; 5.9). Eleven of 14 significant correlations were found between fat fraction and a clinical task in the corresponding muscles (*r* = −0.312 to −0.769, CI = −0.874 to −0.005). At follow-up, fat fractions had increased significantly in 17 of the 26 muscles: mean 1.7% in the upper leg muscles (CI = 0.8–2.4), 1.7% (1.0–2.3) in the lower leg muscles, and 1.9% (0.6–3.3) in the orofacial muscles (*p* < 0.05). The largest increase was seen for the soleus (3.8%, CI = 2.5–5.1). Correlations were found between disease duration and repeat length vs increased fat fraction in 7 leg muscles (*r* = 0.323 to −0.412, *p* < 0.05).

**Discussion:**

According to quantitative muscle MRI, the tongue, adductor magnus and soleus show the largest fat infiltration levels in patients with OPMD. Fat fractions increased in several orofacial and leg muscles over 20 months, with the largest fat fraction increase seen in the soleus. This study supports that this technique is sensitive enough to show worsening in fat fractions of orofacial and leg muscles and therefore a responsive biomarker for future clinical trials.

## Introduction

Oculopharyngeal muscular dystrophy (OPMD) is a rare, autosomal dominant, progressive neuromuscular disease, with an onset usually around the fifth decade.^1^ The early features are ptosis combined with dysphagia, with limb weakness becoming a progressive feature as the disease progresses.^2,3^ OPMD is the result of a trinucleotide repeat expansion in the PABPN1 gene; an alanine expansion of the GCN[10] expansion is pathogenic.^4^ In addition, the size of PABPN1 genotype seems to influence the disease severity and progression. A large French study showed that patients with OPMD with longer expansion of PABPN1 were diagnosed at an earlier age compared with patients with shorter expansions of PABPN1.^5^ There is no curative treatment for OPMD.

Studies in various neuromuscular disorders^6,7^ have used MRI to evaluate muscle alterations, showing that muscle MRI can be a sensitive measure to show disease progression, even before changes in strength and functional tests can be identified.^8^ The most affected muscles in OPMD detected by MRI are the tongue, hamstrings, hip adductor, and calf muscles.^3,9-14^ Most of the studies used semiquantitative scores to determine the amount of fatty infiltration patients with in OPMD. Only one small (semi)quantitative MRI study in patients with OPMD (n = 8) has been published showing changes in muscle fat fraction in 7 of 18 leg muscles over the 13-month follow-up.^10^ This study showed no disease progression in semiquantitative scores. Therefore, studies assessing the natural history in OPMD with more precise measurements and a high sensitivity to change in fat infiltration are needed.

The aim of this study was to describe the pattern of fatty infiltration of orofacial and leg muscles using quantitative muscle MRI in a national large cohort of OPMD and to determine whether MRI can be used as an imaging biomarker of disease progression in OPMD.

## Methods

### Standard Protocol Approvals, Registrations, and Patient Consents

The medical ethics committee (CMO Arnhem-Nijmegen gave approval for the study (NL54606.091.15) and all patients gave their approval by signing their informed consent.

### Patients

Patients with OPMD (older than 18 years) were recruited from the national neuromuscular database (CRAMP database: Computer Registry of All Myopathies and Polyneuropathies)^15^ or by their treating physicians (distributed evenly across the country). We are the recognized national OPMD reference center, part of the “Muscle center of the Netherlands.” In addition, family members of patients with OPMD who enrolled in this study were asked to participate through an information letter to find asymptomatic carriers or patients with subclinical signs of OPMD. All participants gave permission for genetic testing. Exclusion criteria were nonoral food intake and any other cause of dysphagia.

### Muscle MRI Acquisition

Muscle MRI was performed using a scanning protocol as published previously.^16-18^ In short, the head-neck region and the lower extremities were examined with a 3T MRI (TIM Trio, Siemens, Erlangen, Germany). Patients were positioned supine and head first. Imaging stacks were positioned with the most proximal slice at the trochanter major for the upper legs, at the head of the fibula for the lower legs, and the center of the forehead for the orofacial muscles. Axial T1-weighted turbo-spin echo images were collected of the upper and lower legs (repetition time = 646–735 ms, echo times = 12 ms, field of view = 434 × 262 mm, in-plane resolution = 0.97 × 0.97 mm, slice gap/thickness = 5/5 mm, number of slices = 32). Sagittal T1-weighted 3D MP RAGE images with a resolution of 1 × 1 × 1 were collected for the head-neck region to allow semiquantitative assessment of fat infiltration (repetition time = 1,900 ms, echo time = 12 ms, field of view = 250 × 250 mm, in-plane resolution = 0.98 × 0.98 mm, slice thickness/gap = 1/0 mm, number of slices = 192). To quantitatively assess fatty infiltration, we acquired a 3D 2-point Dixon sequence (repetition time = 10 ms, echo times = 2.45 and 3.67 ms, field of view = 299 × 435 mm, in-plane resolution = 1.36 × 1.36 mm, slice thickness/gap = 5/0 mm). The Dixon settings were the same for all 3 regions, with the exception for the number of slices (upper legs/lower legs: 72 slices, head-neck: 64 slices).

### Muscle MRI Analysis

**Figure 1:**
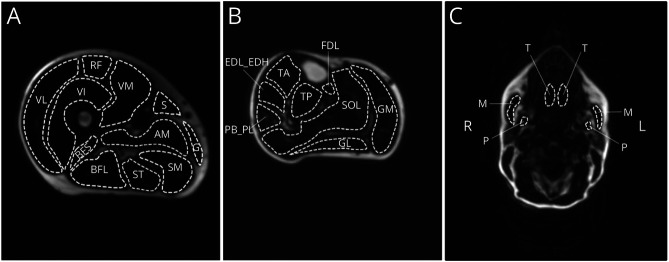
Typical Examples of a Dixon Fat Fraction Image of the Right Upper Leg, Right Lower Leg, and Head-Neck (A) Regions of interest per muscle of the upper leg. AM = adductor magnus; BFL = biceps femoris long head; BFS = biceps femoris short head; G = gracilis; RF = rectus femoris; S = sartorius; SM = semimembranosus; ST = semitendinosus; VI = vastus intermedius; VL = vastus lateralis; VM = vastus medialis. (B) Regions of interest per muscle of the lower leg. EDL_EDH = extensor digitorum/hallucis longus; FDL = flexor digitorum longus; GL = gastrocnemius lateralis; GM = gastrocnemius medialis; PB_PL = peroneus brevis and longus; SOL = soleus; TA = tibialis anterior; TP = tibialis posterior. (C) Regions of interest per muscle of the orofacial muscles. M = masseter, P = pterygoid medialis and T = tongue. R = right, L = left.

A fat fraction map, with voxel values ranging from 0% to 100%, was calculated from the Dixon images. The Dixon sequences acquire in-phase and out-phase images that were reconstructed with the vendors software into fat and water images. These fat and water images were used to create a quantitative fat fraction map using MATLAB (2018a) by dividing fat image by the sum of the fat and water image. Those fat and water images were reconstructed using the scanner software. Regions of interests (ROI's) were drawn around 12 upper leg muscles, 8 lower leg muscles, and 3 orofacial muscles on the Dixon images (Figure 1). The fat fraction map was used to draw the regions of interest (ROI's) of the muscles. Fat fractions were calculated per ROI. Four medical imaging students were trained to draw the ROI's of the leg muscles. These drawn muscles were checked by one investigator (R.K.). All orofacial muscles were drawn by one investigator (R.K.). For the upper legs, ROIs were drawn on 3 slices, being at 1/3, 1/2, and 2/3 of the femur bone, starting from the trochanter major. The mean fat fraction of each muscle was calculated by the mean of the 3 slices. For the lower legs, ROIs were drawn at single slice, that is, the slice with the greatest periphery of the lower leg. The orofacial muscles were also drawn on a single slice, at the height where the inferior alveolar nerve was visible. The intrinsic tongue muscles (transversus and superior longitudinal) were measured together and named as tongue in this paper.

#### Semiquantitative Muscle MRI

A modified version of the Lamminen scale was used to semiquantitatively assess muscle fatty infiltration of the orofacial muscles (temporal, masseter, pterygoid medialis and lateralis, and tongue muscles) on T1-weighted images.^19^ All available images were used to determine the degree of fat infiltration. An experienced musculoskeletal radiologist (J.d.R.) performed all analyses at baseline and follow-up. Fatty infiltration was scored as: “0 = normal, 1 = mild with only traces of fatty infiltration, 2 = moderate with fatty infiltration in less than 50% of the muscle tissue, 3 = severe with fatty infiltration in more than 50% of the muscle tissue, and 4 = the entire muscle replaced by abnormal signal.”^19,20^ We did not analyze the leg muscles with the Lamminen scale because other studies already showed moderate to strong correlations between quantitative muscle fat fractions and semiquantitative Lamminen scores in other neuromuscular diseases.^21,22^ R.K. and J.d.R. analyzed all MRI images at baseline and follow-up blinded for each other's judgments.

### Clinical Measures

To ensure consistency, all measurements were performed by one investigator (R.K.). Muscle strength and functional capacity of the oropharyngeal region and of the upper and lower extremities were measured as described previously^23^ and are explained below in short.

#### Muscle Strength

- For *manual muscle testing*, the scale of the Medical Research Council (MRC) was used, ranging from 0 (no muscle contraction) to 5 (maximal muscle strength), applied to the neck (extension and flexion), elbows (extension and flexion), the knees (extension and flexion), the hips (abduction and flexion), the feet (dorsal flexion and plantar flexion), handgrip, the wrists (extension and flexion), and shoulder abduction bilaterally (score 0–130).^24^
- *Fixed dynamometry (*Newton, N) was performed with the use of the strength transducer KAP-S 2 kN (Angewandte System Technik GmbH) for measuring the maximal isometric contraction of the shoulder abduction (deltoid muscle), the hip flexion (iliopsoas muscle), and the knee extension (quadriceps muscle).
- *Maximum bite force (MBF) of the frontal teeth* was measured with the use of a Bite Force Gauge (Vrije Universiteit, Amsterdam).
- *Maximum isometric anterior tongue pressure (MITP*, kilopascal, kPa*)* was measured with the Iowa Oral Performance Instrument.^25^

For all force measurements, the best performance of 3 trials was used for further analysis.

#### Functional Capacity

- The *timed stair walking test* (10-steps) measured in seconds.^26^
- The *motor function measure (MFM)* consists of 3 parts: D1 includes stance and transfer tasks, D2 includes tasks of axial and proximal muscles, and D3 includes tasks of distal muscles. The total score ranges from 0%–100%, where a score of 100% indicates no functional motor deficits.^27^
- Swallowing and chewing capacity was measured by the *maximum swallowing volume (MSV)*, *maximum swallowing speed (MSS)*, and the *test of masticating and swallowing solids (TOMASS).* The MSV is the maximal amount of water (mL) a patient can swallow in one swallowing attempt.^28^ MSS is the result of drinking 150 mL water as quick as possible.^29,30^ The TOMASS (one part) is the time needed to eat a standardized cracker as fast as possible, in seconds.^31^
- The speech capacity was measured by the *maximum repetition rate (MRR)* and *maximum phonation time (MPT).* The MRR is performed by producing the syllable sequences (/pa/, /ta/, /ka/, /pataka/) as fast as possible (syllables/second), measured during the first 5 seconds.^32^ For the MPT, it requires the phonation of an/a/as long as possible (seconds).^33^

### Statistical Analysis

All statistical calculations were performed with IBM SPSS Statistics (version 25), and *p* values of <0.05 were considered statistically significant. At baseline, the pattern of fatty infiltration (%) was analyzed in each patient in comparison with age to realize a heatmap. The relation between the Lamminen scores and quantitative MRI fat fractions was examined with Spearman rho correlation coefficients. Whether and how strong clinical measures and fat fractions of the corresponding muscles are associated was also tested with Spearman rho correlation coefficients, including 95% confidence intervals. The fat fractions at baseline and follow-up were compared using the paired samples *t* tests with 95% confidence intervals. Apart from these absolute differences, relative changes in fat fractions were calculated in percentages (where the lower and upper limits of the 95% confidence intervals were also converted into percentages). Responsiveness was assessed by calculating the standardized response mean analysis (SRM; mean_change_/SD_change_). Responsiveness is considered small (SRM values of 0.2), moderate (SRM >0.5), or large (SRM >0.8).^34^ The Lamminen scores at baseline and follow-up were compared using the related samples Wilcoxon signed-rank test. The relation between the changes in fat fraction with the changes in clinical measures was tested with Spearman rho correlation coefficients, including 95% confidence intervals. Spearman rho correlation coefficients were also used to test whether the changes in fat fractions (%) are associated with disease duration and repeat length.

### Data Availability

On reasonable request, the anonymized data that support the findings of this study are available from the corresponding author.

## Results

### Patients

**Figure 2:**
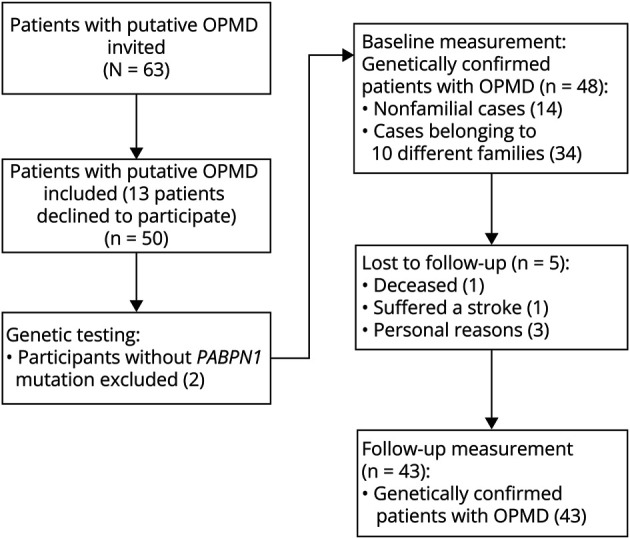
Patient Recruitment and Inclusion of the Study

Forty-four patients with OPMD were recruited through their treating physicians or the national neuromuscular database. In addition, 19 family members of these patients being putative OPMD patients were asked to participate as well. Figure 2 shows the results of the recruitment and inclusion of the study, showing a total of 43 patients with genetically confirmed OPMD participating in this longitudinal study. Ten different families were identified in our study group, geographically distributed approximately evenly across the country.

Table 1 summarizes the demographic and genetic details of the patients, including 4 patients without any complaints at baseline which are presented as asymptomatic carriers. The baseline data were collected from February 2016 until January 2017, and the follow-up data were collected from October 2017 until June 2018. The mean interval between the measurements was 20 months (SD 1.8).

**Table 1:** Patient Characteristics

Patient characteristics (n = 43)	
Mean age at baseline (range)	60.2 (44–79 y)
Sex (men/women)	21/22
Mean age at onset (range)	50.3 (37–73 y)
Repeat length (N)	
11/11	2
10/12	3
10/13	6
10/14	7
10/15	2
10/16	23

Five head-neck scans, 4 upper leg scans, and 6 lower leg scans were missing due to technical errors or patients experienced claustrophobia during the MRI scan.

### Pattern of Fatty Infiltration at Baseline

**Figure 3:**
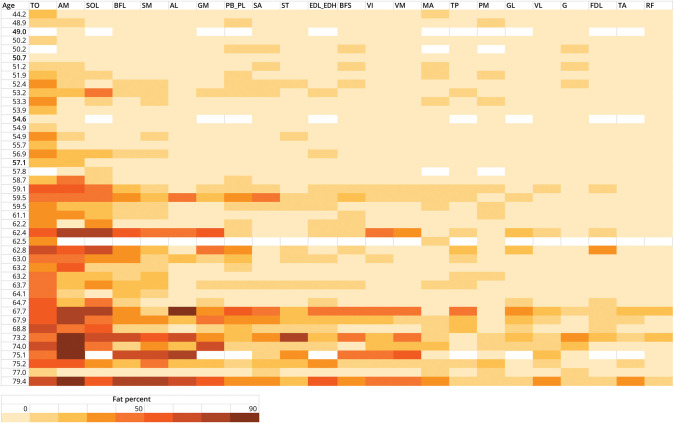
Heatmap Displaying the Pattern of Fatty Infiltration in OPMD at Baseline Each row represents a patient ordered by age. Each column represents a muscle ordered by the degree of fatty infiltration (mean fat fraction of left and right side). AL = adductor longus; AM = adductor magnus; BFL = biceps femoris long head; BFS = biceps femoris short head; EDL_EDH = extensor digitorum longus_extensor hallucis longus; FDL = flexor digitorum longus; G = Gracilis; GL = gastrocnemius lateralis; GM = gastrocnemius medialis; MA = masseter; PB_PL = peroneus brevis_peroneus longus; PM = pterygoid medialis; RF = rectus femoris; SA = sartorius; SM = semimembranosus; SOL = soleus; ST = semitendinosus; TA = tibialis anterior; TO = tongue; TP = tibialis posterior; VI = vastus intermedius; VL = vastus lateralis; VM = vastus medialis. White bar = no MRI scan due to technical errors or claustrophobia. Bold age = asymptomatic carrier.

Fat fractions per muscle ranged from 2% to 89%. Figure 3 shows a heatmap of the fat fractions of all muscles with the participants sorted by age in years (top to bottom) and the muscles sorted from highest average fat fraction to lowest average fat fraction (left to right). The 3 most severely affected muscles were the tongue (mean fat fraction: 37.0%, SD 16.6), the adductor magnus (mean: 31.9%, SD 27.1), and soleus (mean: 27.9%, SD 21.5). The rectus femoris and tibialis anterior muscles were least severely affected (mean fat fractions: 6.8%, SD 4.7% and 7.5%, SD 5.9, respectively). Figure 3 shows the fat fractions of all muscles sorted by the participants' age (years). Furthermore, the heatmap reveals that the severely affected muscles at later disease stages are also the muscles that are affected early in the disease.

#### Correlation Between Fat Fractions and Lamminen Scores at Baseline

The Lamminen score correlated significantly with the fat fraction of the left masseter (*r* = 0.412, *p* = 0.009), the left tongue muscle (*r* = 0.738, *p* < 0.001), and the right tongue muscle (*r* = 0.851, *p* < 0.001). The fat fraction of the temporalis and pterygoid medialis muscles did not correlate significantly with the Lamminen scores (*p* > 0.05).

#### Correlation Between Fat Fractions and Clinical Measures at Baseline

Eleven of 14 correlations between mean fat fractions and corresponding clinical tasks were found to be statistically significant (Table 2).

**Table 2:** Correlations Between Fat Fractions (%) and Clinical Measures at Baseline

Fat fractions (%)	MFM total	MFM D1	MRC sum score	MRC knee extension	MRC knee flexion	Dynamometry knee extension	10-Step stair test
Mean fat fraction, total legs	*r* = −0.722 (−0.847 to −0.519) *p* < 0.001 (n = 39)	*r* = −0.719 (−0.846 to −0.515) *p* < 0.001 (n = 39)	*r* = −0.519 (−0.722 to −0.234) *p* = 0.001 (n = 39)	NA	NA	NA	*r* = 0.509 (0.196 to 0.728) *p* = 0.002 (n = 34)
Mean fat fraction, quadriceps	NA	NA	NA	*r* = −0.644 (−0.796 to −0.415) *p* < 0.001 (n = 42)	NA	*r* = −0.312 (−0.572 to −0.005) *p* = 0.047 (n = 41)	NA
Mean fat fraction, hamstrings	NA	NA	NA	NA	*r* = −0.659 (−0.810 to −0.426) *p* < 0.001 (n = 39)	NA	NA

Fat fractions (%)	MITP	Maximum bite force	Maximum chewing time	Maximum swallowing speed	Maximum swallowing volume
Mean fat fraction, tongue	*r* = −0.769 (−0.874 to −0.596) *p* < 0.001 (n = 40)	NA	NA	*r* = −0.503 (−0.709 to −0.218) *p* = 0.001 (n = 40)	*r* = −0.364 (−0.613 to −0.050) *p* = 0.021 (n = 40)
Mean fat fraction, masseter	NA	*r* = −0.176 (−0.470 to 0.152) *p* = 0.276 (n = 40)	*r* = 0.005 (−0.338 to 0.347) *p* = 0.976 (n = 35)	NA	NA
Mean fat fraction, pterygoid medialis	NA	*r* = −0.456 (−0.677 to −0.159) *p* = 0.003 (n = 40)	*r* = 0.158 (−0.195 to 0.475) *p* = 0.364 (n = 35)	NA	NA

Abbreviations: MFM = motor function measure; MITP = maximum isometric tongue pressure; MRC = medical research council; NA = not applicable.

Mean fat fraction refers to the mean of the left and right side of the muscle. Mean of total legs = mean of muscles of upper and lower legs. Quadriceps = mean of Rectus Femoris, Vastus Intermedius, Vastus Lateralis, and Vastus Medialis. Hamstrings = mean of Biceps Femoris long head, Biceps Femoris short head, Semimembranosus, and Semitendinosus. 95% CI and number of patients are displayed in parentheses.

**Figure 4:**
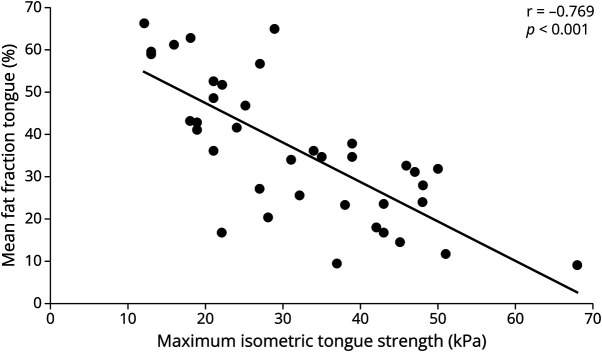
Mean Fat Fraction of the Tongue Muscle Correlates With Maximum Isometric Tongue Strength (kPa)

The mean fat fraction of the total legs correlated significantly with the MFM total, MFM part D1, MRC sum score, and the 10-step stair test. MRC scores of knee extension and flexion correlated significantly with the mean fat fraction of the corresponding muscle groups. The mean fat fraction of the orofacial muscles correlated significantly with the maximum swallowing capacity, maximum isometric tongue pressure, and MBF. The strongest correlation was found between the mean fat fraction of the tongue muscle and the maximum isometric tongue pressure (*r* = −0.769, *p* < 0.001, Figure 4).

### Longitudinal Changes in MRI Outcome Measures

#### Quantitative Fat Fraction

Almost all muscles (25 of 26) showed increased fat fraction from baseline to follow-up, but 17 of the 26 muscles reached statistical significance (Table 3). When even more strictly calculated with Bonferroni correction, only 8 remain with a *p* value <0.001. Per muscle group, an overall increase in absolute fat fraction over time was seen for the orofacial muscles (1.9%, *p* = 0.005), the upper leg muscles (1.7%, *p* < 0.001), and the lower leg muscles (1.7%, *p* < 0.001). Of the orofacial muscles, the right masseter and the tongue muscle showed a significant increase in fat fraction (*p* < 0.05). Fat fraction also increased in most of the upper and lower leg muscles over time. The largest increase in absolute fat fraction was seen for the soleus muscle (3.8%). Responsiveness of fat fraction was the highest for the soleus (SRM = 0.95), followed by the biceps femoris long head (SRM = 0.91) and adductor magnus (SRM = 0.79).

**Table 3:** Mean Fat Fractions (%) and SD at Baseline and Follow-up, Percentage of Worsening (95%), *p* Values and Standardized Response Means of All Orofacial Muscles, Upper Leg Muscles, and Lower Leg Muscles

	Baseline fat fraction Mean (SD)	Follow-up fat fraction Mean (SD)	Absolute change in fat fraction (95% CI)	Relative change in fat fraction (95% CI)	*p* Value	SRM
Orofacial muscles						
Masseter L	10.6 (7.1)	11.6 (8.1)	1.0 (−0.6 to 2.5)	9.4 (−5.7 to 23.6)	0.236	0.19
Masseter R	10.6 (6.1)	12.2 (7.4)	1.6 (0.1 to 3.1)	15.0 (0.9 to 29.2)	0.043	0.35
Pterygoid medialis	9.6 (4.7)	11.4 (9.8)	1.8 (−0.5 to 4.0)	18.8 (−5.2 to 41.7)	0.122	0.26
Tongue	37.0 (17.0)	39.8 (19.2)	2.8 (1.0 to 4.7)	7.6 (2.7 to 12.7)	0.003	0.50
Mean orofacial muscles	19.1 (7.6)	21.0 (9.3)	1.9 (0.6 to 3.3)	9.9 (3.1 to 17.3)	0.005	0.50
Upper leg muscles						
Adductor Longus	13.7 (19.4)	15.8 (21.9)	2.1 (0.4 to 3.8)	15.3 (2.9 to 27.7)	0.015	0.40
Adductor Magnus	30.3 (26.4)	33.6 (27.7)	3.3 (1.9 to 4.6)	10.9 (6.3 to 15.2)	**<0.001**	0.79
Biceps femoris long head	18.1 (16.6)	21.1 (18.0)	3.0 (1.9 to 4.0)	16.6 (10.5 to 22.1)	**<0.001**	0.91
Biceps femoris short head	11.3 (10.5)	13.5 (12.4)	2.2 (0.7 to 3.6)	19.5 (6.2 to 31.9)	0.004	0.49
Gracilis	8.1 (5.8)	8.3 (5.9)	0.2 (−0.4 to 0.8)	2.5 (−4.9 to 9.9)	0.487	0.11
Rectus femoris	6.8 (4.8)	7.0 (4.9)	0.2 (−0.3 to 0.7)	2.9 (−4.4 to 10.3)	0.384	0.12
Sartorius	12.8 (10.5)	13.8 (11.9)	1.0 (0.1 to 2.0)	7.8 (0.8 to 15.6)	0.036	0.33
Semimembranosus	17.5 (15.5)	20.3 (17.1)	2.8 (1.4 to 4.1)	16.0 (8.0 to 23.4)	**<0.001**	0.67
Semitendinosus L	11.5 (10.9)	13.2 (12.0)	1.7 (0.6 to 2.7)	14.8 (5.2 to 23.5)	0.002	0.52
Semitendinosus R	11.4 (11.4)	11.9 (11.4)	0.5 (−0.3 to 1.2)	4.4 (−2.6 to 10.5)	0.243	0.21
Vastus Intermedius	11.0 (10.8)	12.7 (13.5)	1.7 (0.7 to 2.8)	15.5 (6.4 to 25.5)	0.002	0.53
Vastus Lateralis	7.8 (6.1)	8.4 (7.1)	0.6 (0.1 to 1.3)	7.7 (1.3 to 16.7)	0.091	0.29
Vastus Medialis	10.3 (11.1)	11.7 (12.5)	1.4 (0.5 to 2.3)	13.6 (4.9 to 22.3)	0.004	0.50
Mean upper leg muscles	13.2 (10.9)	14.9 (11.9)	1.7 (0.8 to 2.4)	12.9 (6.1 to 18.2)	**<0.001**	0.64
Lower leg muscles						
Extensor Digitorum/Hallucis Longus	13.2 (11.4)	15.1 (13.2)	1.9 (0.8 to 3.0)	14.4 (6.1 to 22.7)	0.001	0.58
Flexor Digitorum Longus	7.4 (4.4)	7.3 (4.5)	−0.1 (−0.8 to 0.7)	−1.4 (−10.8 to 9.5)	0.877	−0.05
Gastrocnemius Lateralis	9.5 (8.0)	12.0 (10.9)	2.5 (1.3 to 3.9)	26.3 (13.7 to 41.1)	**<0.001**	0.68
Gastrocnemius Medialis	15.5 (16.6)	17.5 (17.2)	2.0 (1.1 to 2.9)	12.9 (7.1 to 18.7)	**<0.001**	0.71
Peroneus Brevis/Peroneus Longus	15.2 (10.3)	16.9 (11.8)	1.7 (0.8 to 2.6)	11.2 (5.3 to 17.1)	0.001	0.63
Soleus	28.8 (21.7)	32.6 (23.2)	3.8 (2.5 to 5.1)	13.2 (8.7 to 17.7)	**<0.001**	0.95
Tibialis Anterior L	7.3 (5.4)	7.9 (6.4)	0.6 (−0.1 to 1.4)	8.2 (−1.4 to 19.2)	0.098	0.27
Tibialis Anterior R	7.9 (6.7)	8.6 (6.8)	0.7 (0.1 to 1.4)	8.9 (1.3 to 17.7)	0.034	0.35
Tibialis Posterior	10.7 (8.7)	12.6 (10.7)	1.9 (0.8 to 3.0)	17.8 (7.5 to 28.0)	0.001	0.59
Mean lower leg muscles	13.7 (9.6)	15.4 (11.0)	1.7 (1.0 to 2.3)	12.4 (7.3 to 16.8)	**<0.001**	0.89

Abbreviation: SRM = standardized response mean.

When no significant difference is present between left and right side, the muscle is represented in mean. *p* values in bold are statistically significant after Bonferroni correction.

As previously published,^23^ in this cohort, 9 clinical measures changed over time (strength of the deltoid muscle, quadriceps, iliopsoas and tongue, plus the MRC sum score, the 10-step stair test, MFM part D1, MFM total score, and the maximum repetition rate of/pa/). However, when comparing changes in fat fraction with these changes in clinical measures, we only found a significant correlation between the deterioration of mean fat fractions of the total legs with the decrease of the MFM total score (*r* = −0.447, *p* = 0.006) and with part D1 of the MFM (*r* = −0.435, *p* = 0.007).

#### Lamminen Scale

The Lamminen score increased significantly over 20 months' time for the left masseter (baseline: median 2.0, range 1–2; follow-up: median 2.0, range 1–3; *p* < 0.046), left pterygoid medialis (baseline: median 2.0, range 1–2; follow-up: median 2.0, range 1–3; *p* < 0.025), and right tongue muscles (baseline: median 2.0, range 1–4; follow-up: median 2.0, range 1–4; *p* < 0.046), but not for the other orofacial muscles.

### Asymptomatic Carriers

At baseline, one asymptomatic carrier showed elevated fat fractions in the adductor magnus (mean fat fraction of left and right side: 20.4%) and tongue (24.1%). The fat fraction of the adductor magnus was at the follow-up 20.7% and 26.7% for the tongue. At follow-up, fat fraction had increased from 8.6% to 10.9% for the biceps femoris longus and from 9.4% to 14.0% for the semimembranosus. The other 3 asymptomatic carriers presented a fat fraction in the adductor magnus of 5.8%, 6.3%, and 4.6%. In the tongue, 2 asymptomatic carriers presented a fat fraction of 8.9% and 9.3%; one asymptomatic carrier had a missing value for the fat fraction in the tongue. The muscles in these 3 asymptomatic carriers all had fat fractions less than 10%.

### Natural History

There was a significant, but small correlation between disease duration (y) and change in fat fraction (%) for the biceps femoris longus (*r* = 0.323, *p* = 0.045), vastus intermedius (*r* = 0.337, *p* = 0.036), and extensor digitorum/hallucis longus (*r* = 0.331, *p* = 0.046) muscles. Repeat length showed a significant, but small negative relation with change in fat fraction (%) for the biceps femoris short head (*r* = −0.339, *p* = 0.037), extensor digitorum/hallucis longus (*r* = −0.412, *p* = 0.011), gastrocnemius lateralis (*r* = −0.352, *p* = 0.038), gastrocnemius medialis (*r* = −0.337, *p* = 0.041), and peroneus brevis/peroneus longus (*r* = −0.392, *p* = 0.016) muscles. No relation was found between these patient characteristics (disease duration or repeat length) and the change in fat fraction (%) for the orofacial muscles and other upper and lower leg muscles (*p* > 0.05).

## Discussion

This longitudinal quantitative muscle MRI study shows that progression of fat infiltration can be detected in the orofacial and lower extremity muscles in patients with OPMD within a period of 20 months. At baseline, the rectus femoris and tibialis anterior muscles were least affected by fat infiltration, while the tongue, the adductor magnus, and the soleus muscles showed the highest levels of fat infiltration, which is consistent with the international study by Alonso-Jimenez et al.^12^ Almost all muscles (25 of 26) showed an increase in fat fraction over time, 17 of 26 orofacial and lower extremity muscles showed a statistical significant increase in fat fraction from baseline to follow-up, with the largest increase seen in the soleus muscle (3.8%). A progression in fat fraction over time was detected in all muscle groups, for example, the upper leg, lower leg, and orofacial muscles. In addition, fat infiltration was also detected with the semiquantitative Lamminen score in the masseter, pterygoid medialis, and tongue muscles. These findings confirm that muscle MRI can be used as a longitudinal biomarker in OPMD within a timeframe of 20 months.

Longitudinal muscle MRI studies are still rare in OPMD. There is only one other study consisting of 8 patients showing that 39% (7/18) of the leg muscles worsened over 13 months. In our study with 43 patients, 65% (17/26) of the orofacial and leg muscles worsened, being nearly twice as much, which can also be explained by the longer follow-up period of 20 months and the larger sample size.

In this study, muscle MRI correlates strongly with clinical measures. Similar findings were reported by our group on facioscapulohumeral muscular dystrophy and supports MRI as biomarker for disease severity.^16^ A significant correlation between the MRI quantitative and semiquantitative assessments was only seen for the right masseter and tongue muscles. No correlation was found for the left masseter, temporal, and pterygoid medialis muscles, probably because the Lamminen score is less sensitive to detect small changes in fatty infiltration.^35^ Other MRI studies in OPMD showed moderate to strong correlations between fat fractions and Lamminen scores of leg muscles.^21,22^ Further research is needed to assess the fatty infiltration and the relationship between fat fractions and Lamminen scores of the orofacial muscles to determine whether the Lamminen score is a sensitive outcome measure to detect fatty infiltration in orofacial muscles too. We found limited significant correlations between the changes in fat fraction with changes in clinical measures over time. A possible cause for this finding is that the changes over time are not that large yet. However, we were able to find significant changes over time with clinical measures, quantitative muscle ultrasound, and now with muscle MRI.^23,36^ In this study, the leg muscles showed the largest deterioration and the highest responsiveness, for example, the soleus muscle. Further longitudinal research is needed to better understand the correlation between increasing fat fractions and worsening clinical performances to observe which measure is the most sensitive in designing future clinical trials. Our results showed that change in fat fraction of several leg muscles had a weak, although statistically significant relation with disease duration and repeat length, but clinically only relevant when larger cohorts of patients have settled this point. Patients with OPMD with a long disease duration showed larger changes in fat fractions compared with patients with OPMD with shorter disease durations. However, we did not expect the results, and this is in contrast with our previous findings,^23,36^ where fast and slow rates of disease progression, according to clinical and ultrasound measurements, were found in patients with short and with long disease durations and irrespective of repeat lengths. Obviously, larger research groups are needed to better understand how disease duration influences disease progression in OPMD.

Remarkably, the rectus femoris muscle appeared normal on MRI, while it was one of the most affected muscles in our muscle ultrasound study.^36^ In addition, no significant deterioration was seen for the rectus femoris muscle. A previous study has shown that MRI detects fatty infiltration and edema, but not fibrosis, while ultrasound does.^21^ This suggests that muscle involvement in OPMD starts before fatty infiltration is detectable on MRI.

Unique in this study is that we were able to include some asymptomatic carriers. One of the 4 asymptomatic carriers already presented elevated fat fractions (adductor magnus 20.4% and tongue 24.1%) compared with fat fractions of less than 10% found in lower extremity muscles of healthy controls.^22,37^ The normal fat fraction levels of the bulbar muscles are less examined and might show more variation in healthy controls.^38^ Both the adductor magnus and tongue fat fractions levels increased at follow-up. This emphasizes the ability of muscle MRI to detect structural muscle changes and disease progression before functional decline and justifies the need for further research with larger groups of asymptomatic carriers and healthy controls.

Although highly important for swallowing, no pharyngeal muscles were assessed because these small muscles were too hard to detect on the Dixon fat fraction images due to the level of resolution. Meanwhile, MRI scanners have been updated and now have improved sequences with higher resolutions, which will make it possible to study the pharyngeal muscles in the future.^39^ In addition, only one slice of the head-neck region was analyzed, while observing more slices through the whole head-neck region would give more insights in the fatty infiltration of the muscles of the head and neck area. Nevertheless, this study gives insight in the pattern of fatty infiltration of the orofacial muscles over time. Discrepancy between muscles when comparing both sides was observed, for example, in the masseter muscle. The left masseter showed increase as well, however not large enough to gain statistical significance. This may be due to natural left-right differences in people (e.g. a favorite chewing side) but also to the sample size. Furthermore, owing to the enrollment of family members who lived together in their childhood, environmental factors cannot be ruled out. However, these are probably small in comparison with genetic and possible epigenetic factors. Finally, although asymptomatic carriers are difficult to identify and recruit, further research with larger groups of asymptomatic carriers is needed to confirm our findings and better understand this disease and its consequences from the start.

In conclusion, this study demonstrates the pattern of fatty infiltration in the orofacial and lower extremity muscles in patients with OPMD and the progression of this fat infiltration within 20 months. Quantitative muscle MRI is a sensitive biomarker that might be useful for future clinical trials of sufficient duration.

## Appendix Authors

**Table:**

Name	Location	Contribution
Rosemarie H.M.J.M. Kroon, MA	Radboud University Medical Center, The Netherlands	Acquisition, analysis and interpretation of data, drafting of the manuscript and tables/figures
Johanna G. Kalf, PhD	Radboud University Medical Center, The Netherlands	Study concept and design, acquisition, analysis, interpretation of data, revision of the manuscript
Bert J.M. de Swart, PhD	Radboud University Medical Center, The Netherlands	Study concept and design, revision of the manuscript
Linda Heskamp, PhD	University Medical Centre Utrecht, The Netherlands	Setting up MR protocol and MR analysis software, revision of the manuscript
Jacky W.J. de Rooy, MD	Radboud University Medical Center, The Netherlands	Analysis and interpretation of data, revision of the manuscript
Baziel G.M. van Engelen, MD, PhD	Radboud University Medical Center, The Netherlands	Grant writing, study concept and design, interpretation of data, revision of the manuscript
Corinne G.C. Horlings, MD, PhD	Radboud University Medical Center, The Netherlands Medical University of Innsbruck, Austria	Study concept and design, analysis and interpretation of data, revision of the manuscript

## Glossary

MFM: motor function measure
MPT: maximum phonation time
MRC: Medical Research Council
MRR: maximum repetition rate
MSS: maximum swallowing speed
MSV: maximum swallowing volume
OPMD: oculopharyngeal muscular dystrophy
ROI: regions of interest
SRM: standardized response mean
TOMASS: test of masticating and swallowing solids
